# Supplementary material for: Strong Opponent of Walnut Anthracnose—Bacillus velezensis and Its Transcriptome Analysis
Source: Microorganisms. 2023 Jul 26;11(8):1885. doi: 10.3390/microorganisms11081885 (PMC10456653; doi:10.3390/microorganisms11081885)
Supplement: Supplementary file 1 [file microorganisms-11-01885-s001.zip › microorganisms-2490309-supplementary.pdf]

Table S1. Methods for treating detached walnut leaves with different concentrations of *B. velezensis* fermentation liquid.

Time \ Treatment / Groups	CK	T_1	T_10	T_50	T_100	T_300	T_600	T_1200
1d	LB	BV_1	BV_10	BV_50	BV_100	BV_300	BV_600	BV_1200
2d	LB	LB	LB	LB	LB	LB	LB	LB
3d	LB	BV_1	BV_10	BV_50	BV_100	BV_300	BV_600	BV_1200
4d	LB	LB	LB	LB	LB	LB	LB	LB
5d	LB	BV_1	BV_10	BV_50	BV_100	BV_300	BV_600	BV_1200
6d	LB	LB	LB	LB	LB	LB	LB	LB
7d	LB	BV_1	BV_10	BV_50	BV_100	BV_300	BV_600	BV_1200
8d	LB	LB	LB	LB	LB	LB	LB	LB
9d	LB	BV_1	BV_10	BV_50	BV_100	BV_300	BV_600	BV_1200
10d	LB	LB	LB	LB	LB	LB	LB	LB
11d	LB	BV_1	BV_10	BV_50	BV_100	BV_300	BV_600	BV_1200
12d	LB	LB	LB	LB	LB	LB	LB	LB
13d	LB	BV_1	BV_10	BV_50	BV_100	BV_300	BV_600	BV_1200
14d	LB	LB	LB	LB	LB	LB	LB	LB
15d	Record experimental results							

Note: 'LB' refers to the spraying of LB liquid culture medium. 'BV_1' to 'BV_1200' denote the spraying of *B. velezensis* fermentation liquid, diluted respectively 1, 10, 50, 100, 300, 600, and 1200 times.

Table S2. Methods for treating detached walnut leaves with different frequency of *B. velezensis* fermentation liquid.

Time \ Treatment / Groups	CK	T1	T2	T3	T4	T5
1d	LB	BV_50	BV_50	BV_50	BV_50	BV_50
2d	LB	BV_50	LB	LB	LB	LB
3d	LB	BV_50	BV_50	LB	LB	LB
4d	LB	BV_50	LB	BV_50	LB	LB
5d	LB	BV_50	BV_50	LB	BV_50	LB
6d	LB	BV_50	LB	LB	LB	BV_50
7d	LB	BV_50	BV_50	BV_50	LB	LB
8d	LB	BV_50	LB	LB	LB	LB
9d	LB	BV_50	BV_50	LB	BV_50	LB
10d	LB	BV_50	LB	BV_50	LB	LB
11d	LB	BV_50	BV_50	LB	LB	BV_50
12d	LB	BV_50	LB	LB	LB	LB
13d	LB	BV_50	BV_50	BV_50	BV_50	LB
14d	LB	BV_50	LB	LB	LB	LB
15d	Record experimental results					

Note: 'LB' represents treatment with sprayed LB liquid medium. 'BV_50' denotes treatment with a 50-fold dilution of *B. velezensis* fermentation broth.

Table S3. Thirty Candidate validation genes for qRT-PCR.

Comparison Group	Gene Name	log2FoldChange	Description	Primer sequence 5′-3′(F;R)
D2_DvsD2_CK	AAV34_RS11010	1.776131137	dipicolinate synthase subunit B	ACAGGCTTTGAGGCGATTGA, TGTCGAAATTCCGAGCACGA
	AAV34_RS05350	1.499983627	nucleoside triphosphataseYtkD	GTGGCTTTTGACTGAGCACG, CGGCACCGGTTTCTTCTTTC
	AAV34_RS09465	2.692174747	glycosyltransferase	ACAAAACGCGCTTGATGCTT, CTTTCACCAGTTCAACGCGG
	AAV34_RS09945	1.367318874	pyrroline-5-carboxylate reductase	GGAGCAGGATCAATGGCTGA, CTCTGTATTGCGGCGGTTTG
	AAV34_RS19815	1.641796073	hypothetical protein	GGAGCCGTTCAGTGCTTCTA, AAATGTTTCTCGGCGCTTCC
	AAV34_RS04950	-0.772224414	glucose-6-phosphate isomerase	AGCTCTTCGGCGAAAGTGAA, TGTCAGCTCATGCTTCGGTT
	AAV34_RS17620	-1.360674783	surfactin non-ribosomal peptide synthetase SrfAA	TGGACAAGGCGGGCATTTAT, CGGCTCAATCGCACTGAATG

	AAV34_RS03595	-1.136964401	ABC transporter permease	CGGAATTGTGACAGCAAGGC, CCGAGTCCCATGACAAGCAT
	AAV34_RS13375	-0.825632821	6-phospho-beta-galactosidase	CCACAGCCACCAACCAGTAT, TCTGAGTCACTGGCGGGATA
	AAV34_RS05540	-1.375134741	sugar ABC transporter permease	TGCCCGTCATTATCAGTCCG, TTTCGGAATGCTTTGCAGCC
D4_DvsD4_CK	AAV34_RS06295	-1.451292447	glycolate oxidase subunit GlcD	AACCGAGGAAGAGGCATTCG, CTGGCAATTTGCGATCTCGG
	AAV34_RS15365	-1.213783547	2-oxo acid dehydrogenase subunit E2	ATTCTCGGAGTAGGCGCAAG, CAGCGCTTTGACCGTTTTCA
	AAV34_RS05485	-3.636324429	ABC transporter ATP-binding protein	TTCCGGACGTGTGGTTTTCA, CGCCCAATACACTTTGCGTT
	AAV34_RS05480	-2.674128086	sensor histidine kinase	CAGACGTTCACAGTGACGGA, CCGGCCGTTTTGTTCATAGC
	AAV34_RS17340	2.12219226	mannitol-1-phosphate 5-dehydrogenase	ATATACCGTGGAGCTTGCCG, AGAAGACGTTCTGCGCTTCA
	AAV34_RS16480	-1.475385613	NUDIX hydrolase	AGGAGTCGGGAATCGACGTA, GTTTCCGTTCCCTCCCTGAG
	AAV34_RS14425	0.82504225	ABC transporter ATP-binding protein	GGGCTGTACCCGAAAGTGAA, ACGGCCGAAATGAACTGGAT
	AAV34_RS03925	0.728063488	ABC transporter ATP-binding protein	CGGAACAGCTTACTGTCGGT, GAACCGTCGATTTTCCGCAG
	AAV34_RS14810	1.563981249	cold-shock protein	TGCTATTCAAGGCGAAGGCT, CGCGGTTTCCTTCAACGATT
	AAV34_RS03870	0.927503887	molybdate ABC transporter permease subunit	TGTTTATGCTCCCGCTCGTT, TACATAAGCGGGAAGGCGAC
D6_DvsD6_CK	AAV34_RS02470	4.101789208	ribose ABC transporter substrate-binding protein RbsB	TCACGATCGCGGCATTATCA, ATGACCTTCATGCCCCGTTT
	AAV34_RS02475	3.118068008	ribose ABC transporter permease	TGATCTCTCTGTCGGAGCCA, GAACGGCGCCATTTTTCCTT
	AAV34_RS02480	3.485194692	sugar ABC transporter ATP-binding protein	GAGAGCCGGAGAAATCCTCG, CTCCGTAATCAGGCCGAGTC
	AAV34_RS01180	2.444323405	PTS sugar transporter subunit IIB	GGTGTCAGGCGATTCAGTCA, ATTTCAGAACCTCCGCTCCG
	AAV34_RS01015	1.412314847	ABC transporter ATP-binding protein UgpC	ACGGCCGAAATGAACTGGAT, ATTTGACAGCGGCTCATCCA
	AAV34_RS14190	-0.556670539	AMP-binding protein	TCGTGAACAGCCCGATGAAA, TCATCAACCGTCATCCAGCC
	AAV34_RS09750	-0.570507603	aldehyde dehydrogenase family protein	ATGACCTTCATGCCCCGTTT, AAGGAGCGGGAAGTTCCAAG
	AAV34_RS13120	-2.219562401	sigma-70 family RNA polymerase sigma factor	GGAGGAAGCCGATGAAACGA, TGATAAGCGTCTCTGCGGTC
	AAV34_RS15795	-1.712733207	FAD-dependent oxidoreductase	CTGTCCGCGATCAAATGCTG, TTTTTCAGAGAGCGGCACGA
	AAV34_RS11640	-1.292494781	dihydroorotase	TGAAGCGGTACATATCGCCC, GTCTCTTACGGCTCTGACGG
Reference gene	*GAPDH*	-	glyceraldehyde-3-phosphate dehydrogenase	CCGCTCGTGTCTTCGGATTA, AACGACCCGGCAGGAATATC

Table S4. Quality evaluation of sequencing datas.

Sample	Raw reads	Clean reads	Clean bases	Q20/%	Q30/%	GC content/%
D2_CK1	7749828	7556388	1.1G	98.5	95.27	47.35
D2_CK2	7886356	7715706	1.2G	97.92	93.84	47.25
D2_CK3	7635178	7425270	1.1G	97.65	93.35	48.21
D2_D1	7843212	7627662	1.1G	97.79	93.58	47.25
D2_D2	7964034	7805838	1.2G	97.73	93.4	46.98
D2_D3	7604142	7381998	1.1G	97.85	93.67	46.98
D4_CK1	7892718	7749264	1.2G	98.3	94.79	48.03
D4_CK2	7679296	7426024	1.1G	98.35	95.07	54.86
D4_CK3	8216340	7980974	1.2G	98.39	95.06	48.36
D4_D1	7932596	7783232	1.2G	98.56	95.43	50.86
D4_D2	7878194	7722002	1.2G	98.17	94.49	49.3
D4_D3	7841320	7592930	1.1G	98.37	95.1	52.42
D6_CK1	7788306	7674686	1.2G	97.75	93.49	47.24
D6_CK2	8474554	8327564	1.2G	98.27	94.78	47.37
D6_CK3	7618120	7517886	1.1G	98.4	95.04	47.47
D6_D1	7820212	7637766	1.1G	97.6	93.28	47.57
D6_D2	7762248	7658784	1.1G	97.74	93.44	47.34
D6_D3	7924090	7799950	1.2G	97.7	93.38	47.04
